# Seven-year experience (2018–2025) of a hospital-based cardiovascular tissue bank in Israel: operational insights and clinical impact

**DOI:** 10.1007/s10561-026-10230-6

**Published:** 2026-06-05

**Authors:** Alina Levy, Helit Cohen, Michael Groysman, Alexandra Natanilov, Meytal Neeman-Azulay, Ehud Raanani, David Mishali, Rachel Kornhaber, Michelle Cleary, Ayelet Di Segni

**Affiliations:** 1 Sheba Tissue and Cell Bank, Sheba Medical Center, 52621 Tel Hashomer, Israel; 2 Cardiology Division, Sheba Medical Center, 52621 Tel Hashomer, Israel; 3 Advanced Biotherapy Center, Sheba Medical Center, 52621 Tel Hashomer, Israel; 4 School of Health - Nursing, University of New England, Armidale, Australia

**Keywords:** Cardiovascular tissue bank, Validation, Cardiovascular tissue procurement

## Abstract

Cryopreserved human heart valves and cardiovascular tissues (homografts) are essential for the surgical treatment of congenital and acquired valvular diseases, particularly in pediatric patients, where prosthetic options are limited. To address the clinical demand for high-quality grafts in Israel, Sheba Medical Center, the largest tertiary care hospital in Israel, established a Good Manufacturing Practice (GMP) compliant Cardiovascular Tissue Bank within the public health system. This report presents an overview of the bank’s operation within the Israeli public health system over seven years, covering donor selection criteria, tissue processing procedures, microbiological safety measures, cryopreservation and distribution, as well as protocol optimization and validation. Between 2018 and early 2025, the tissue bank successfully transitioned from basic sterile techniques to full GMP-grade cleanroom operations, implementing validated protocols for decontamination, packaging, and cryopreservation. During this period, 142 donor hearts were processed, yielding 390 cardiovascular tissues, of which 338 were approved for clinical use and distributed to cardiac surgery centers across Israel. This paper provides a comprehensive operational overview of the Sheba Cardiovascular Tissue Bank from 2018 to 2025, describing protocol refinement, tissue procurement, processing, preservation, regulatory compliance, quality control, and distribution. By combining clinical collaboration, rigorous quality control, and GMP-based infrastructure, the bank plays a pivotal role in ensuring the availability of high-quality cryopreserved homografts for Israeli patients, especially pediatric cases with complex congenital heart disease, and provides a sustainable model for hospital-based cardiovascular tissue banking.

## Introduction

Cryopreserved human heart valves and other cardiovascular tissues, also known as homografts, play an indispensable role in cardiac surgery, particularly for patients with congenital heart defects, infective endocarditis, or complex reoperations (Skific et al. 2023). In pediatric patients, homografts are often preferred due to their superior hemodynamic performance, low thrombogenicity, and the avoidance of long-term anticoagulation treatment (Avşar et al. 2025). However, the availability of high-quality homografts remains a challenge worldwide, exacerbated in regions with underdeveloped tissue banking infrastructure and reliance on imported grafts (Schmiady et al. 2024).

In 2018, Sheba Medical Center, the largest tertiary care hospital in Israel, established a dedicated cardiovascular tissue bank to address national demand for cryopreserved homografts. The Sheba Cardiovascular Tissue Bank operates as a non-profit establishment under the strict regulation of the Israeli Ministry of Health, in accordance with national legislation governing organ and tissue transplantation. Its goal is to provide timely, safe, and size-matched grafts for pediatric and adult patients undergoing valve replacement surgeries, with a particular focus on congenital cardiac surgery.

The Sheba Cardiovascular Tissue Bank procures, processes, stores, and distributes cardiovascular tissue from tissue and organ donors and explanted hearts, operating in compliance with the Israeli Transplantation Law and adhering to international standards (American Association of Tissue Banks 2024; European Directorate for the Quality of Medicines & HealthCare 2022). Since its establishment, the bank has undergone substantial infrastructure development, transitioning from basic sterile processing in operating rooms to a dedicated GMP compliant cleanrooms facility.

**Fig. 1:**
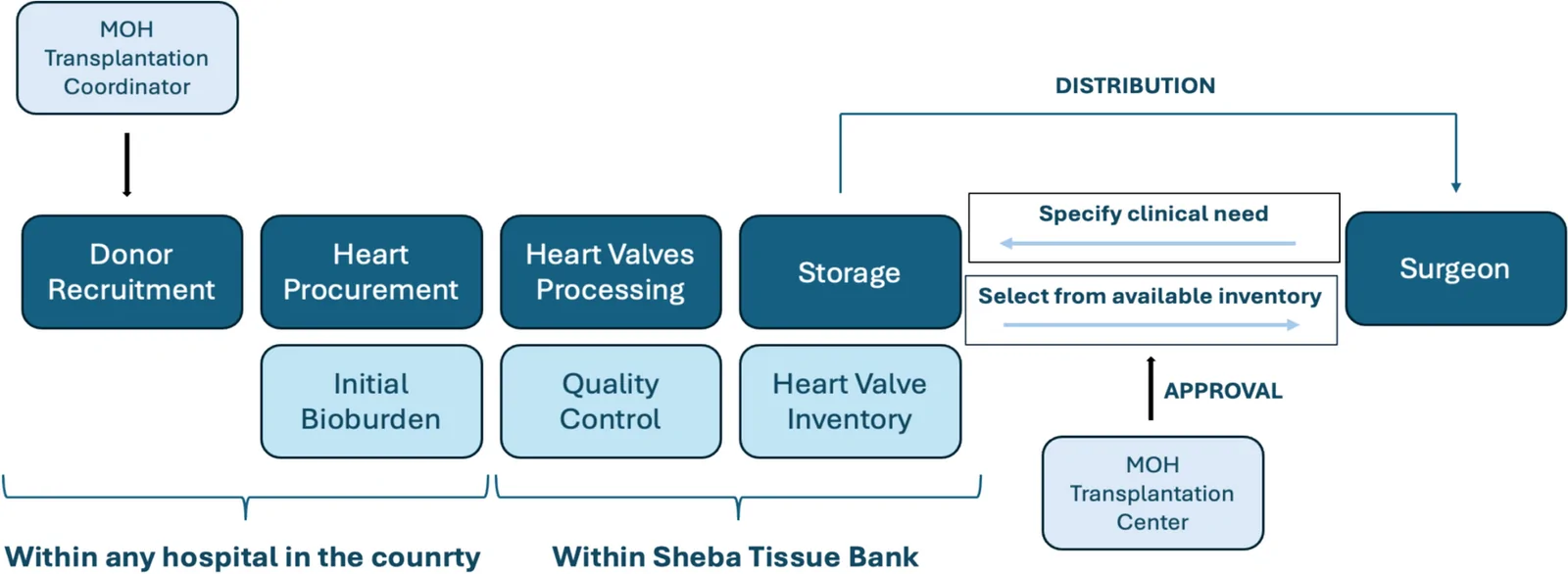
Framework of the Sheba Cardiovascular Tissue Bank. The Sheba Cardiovascular Tissue Bank operates in collaboration with the Israel Ministry of Health and hospitals across the country. An overview of its operational workflow and the interface between the various institutions is illustrated

This report presents an overview of the bank’s operation within the Israeli public health system (Fig. 1) over seven years, covering donor selection criteria, tissue processing procedures, microbiological safety measures, cryopreservation and distribution, as well as protocol optimization and validation. By sharing this experience, we aim to contribute to the growing body of knowledge on cardiac tissue banking and present a practical model for cardiovascular tissue banking in similar healthcare settings.

## Methods

### Ethical statement

This study was conducted in accordance with ethical guidelines for the use of human tissues (SMC-D-2849-25).

### Donor identification

Cardiovascular tissues are procured from donors ranging in age from newborns to 65 years, under one of three conditions: (1) Brain death: brain-dead multi-organ donors when the heart itself is unsuitable for transplantation; (2) Cardiac death; (3) Living heart transplant recipients: cardiovascular tissues are obtained during heart explantation procedures, when morphology and function meet acceptance criteria.

Potential tissue donors are identified through a nationwide network of hospital-based transplant coordinators. Following identification, a transplant coordinator performs an initial evaluation, including review of medical history, assessment of behavioral risk factors, and initiation of serological testing, as detailed below, and then approaches the next of kin to obtain legally required informed consent. At this stage, the cardiovascular tissue bank person of contact is notified, and procurement is scheduled.

### Donor evaluation and eligibility

Donor evaluation and eligibility follow national and international standards (American Association of Tissue Banks 2024; European Directorate for the Quality of Medicines & HealthCare 2022), through comprehensive screening. All donors included in the study were Israeli residents. This process includes medical history review, physical examination to identify signs of infection, malignancy, or high-risk behaviors, serological testing for HIV, HBV, HCV, and syphilis, and assessment of epidemiological risk factors.

Donors are excluded for conditions compromising tissue safety or quality, such as: systemic infections (e.g., sepsis, bacteremia), positive serology, hematological malignancies or most solid tumors, active endocarditis or myocarditis, prion diseases, unknown cause of death, thoracic contamination, or non-sterile conditions. Tissues are also excluded if procurement exceeds 6 h post-mortem at room temperature, or 24 h if refrigerated.

### Additional tests

When HBV core antibody is positive and antigen is negative, the presence of the virus is confirmed by PCR. Negative results qualify the tissue donation. During the summer months (June through October), PCR is performed to identify West Nile Virus. Positive results disqualify the tissue donation. All findings are documented in standardized forms.

### Procurement

The bodies of tissue donors are transferred either directly or after refrigeration to an operating room of the hospital in which death was determined. Tissue procurement is performed exclusively in an operating room under strictly aseptic conditions by trained tissue bank personnel, in collaboration with the full operating room team, including sterile scrub nurses, circulating nurses, and the hospital transplant coordinator.

**Fig. 2:**
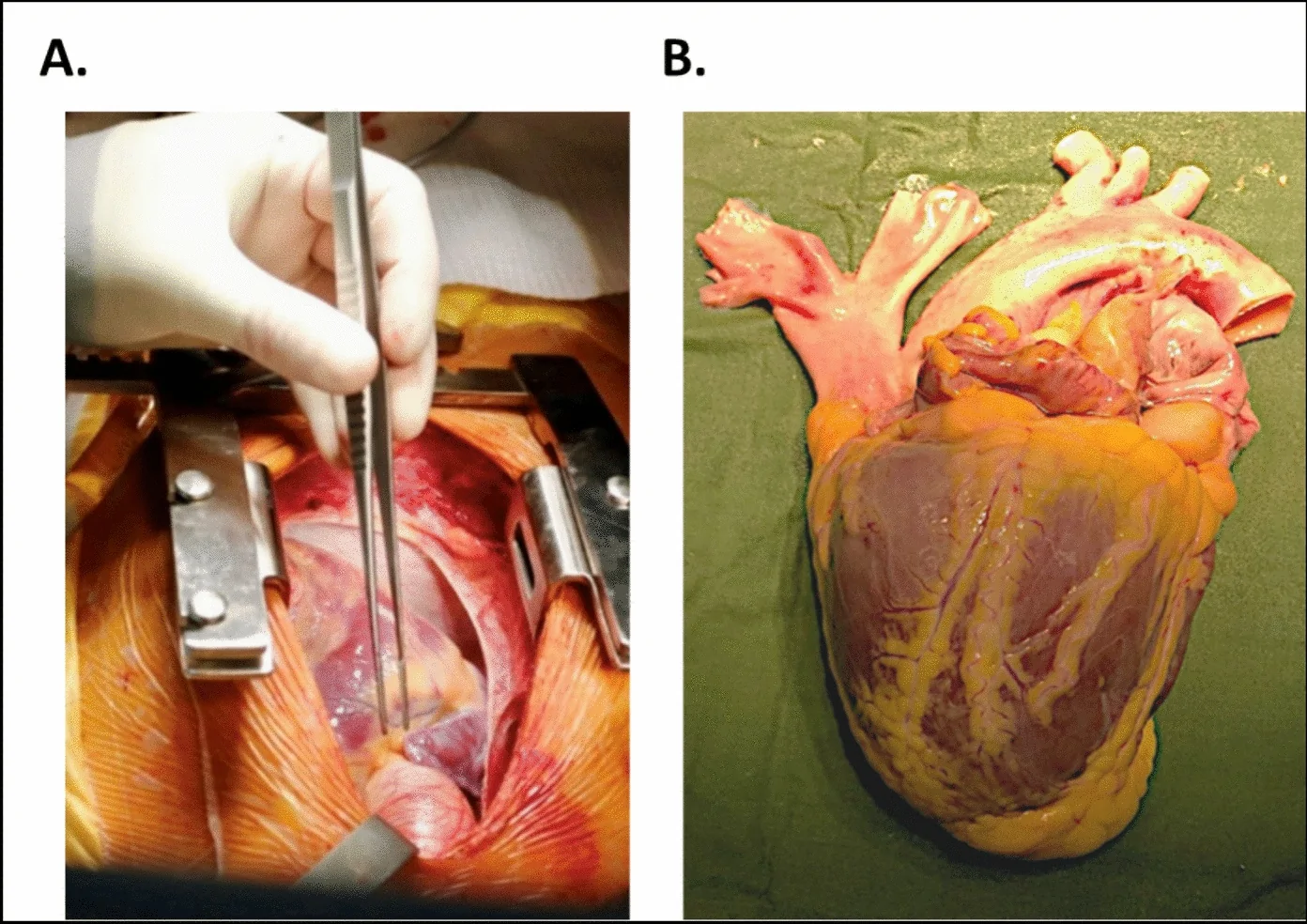
Heart recovery. **A** Aseptic surgical conditions are maintained using the midline sternotomy approach, which offers optimal exposure of the heart for extraction while ensuring the preservation of adequate lengths of the main pulmonary artery, its branches, and the ascending aorta. **B** The heart morphology after recovery and before valve separation

The heart is retrieved using a midline sternotomy approach to preserve anatomical structures (Fig. 2). The procured heart is taken out of the pericardial cavity and flushed with saline after opening the ventricles at the apical level in order to remove all blood and the clots from it. The heart is then placed in a sterile triple bag filled with 500 ml of Ringer’s or saline (0.9% NaCl) solution, which is transported in a container with wet ice. Upon arrival at the tissue bank, the bag containing the heart is stored at 4 °C in a monitored refrigerator.

### Cardiovascular tissue processing

Dissection is initiated within 24 h of procurement. All processing procedures are performed in a GMP-compliant cleanroom (Class A environment with Class C background), ensuring strict aseptic conditions throughout the process.

Before valve separation, the heart is sampled using an eSwab to assess the initial bioburden. It undergoes a systematic external inspection to assess overall morphology, presence of calcifications, structural abnormalities, or signs of infection. Tissues will be discarded if testing positive for pathogens listed by the European Directorate for the Quality of Medicines or showing structural deficiency.

**Fig. 3:**
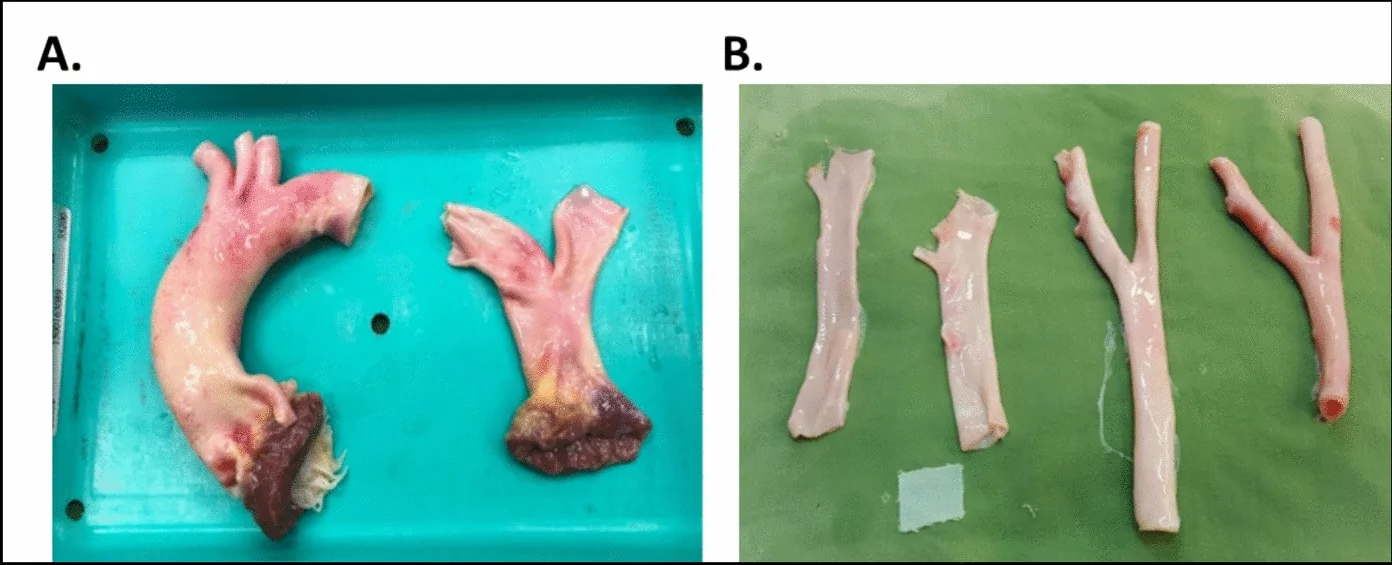
Cardiovascular tissues after separation. **A** Morphology of the aortic valve (left) and pulmonary valve (right) after the dissection process. **B** Blood vessels obtained from the dissection of the iliac artery

The dissection process involves the separation of the aortic and pulmonary roots using standardized surgical techniques (Fig. 3). Surrounding tissues are carefully trimmed to expose the great vessels while preserving adequate lengths of the ascending aorta and pulmonary artery. Special attention is given to preserving valve integrity, including the annulus, commissures, and leaflet structure. Each homograft is handled individually to prevent mechanical damage and cross-contamination.

**Fig. 4:**
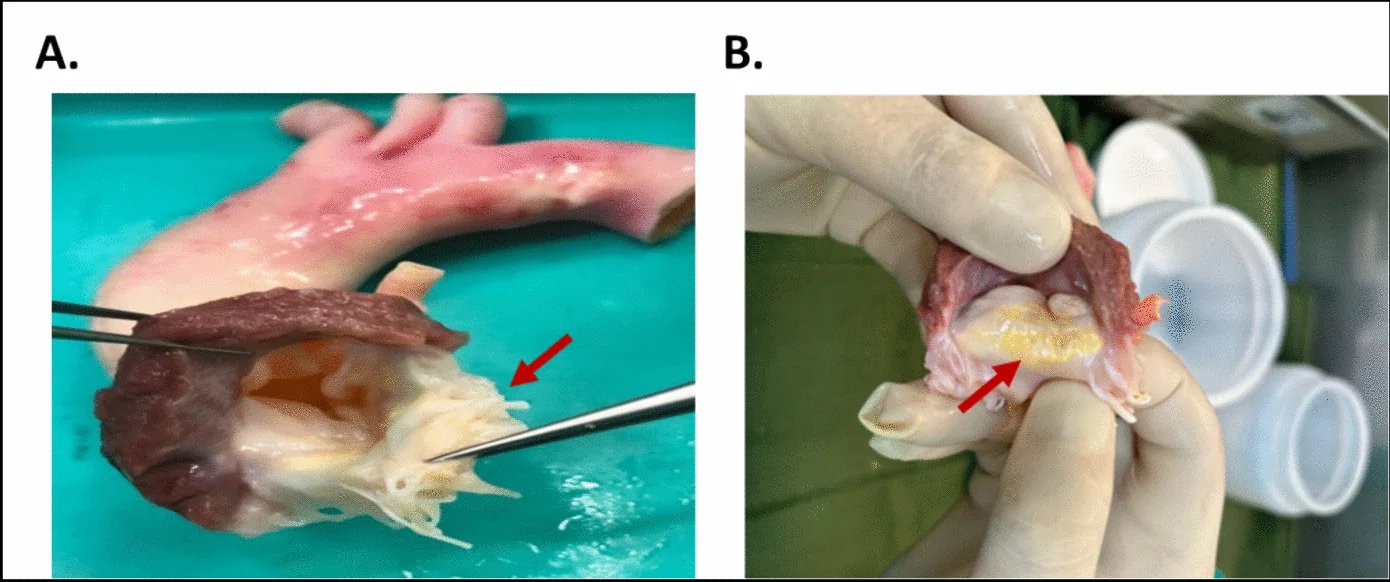
Quality assurance of the dissected valves. **A** Aortic valve morphology after dissection. The arrow indicates the valve’s leaflets. **B** Arrow indicates calcifications of the aortic valve leading to its disqualification

Following dissection, each homograft undergoes detailed morphological evaluation, including: leaflet integrity and mobility, presence of calcifications or fibrosis, structural symmetry and anatomical completeness (Fig. 4).

**Fig. 5:**
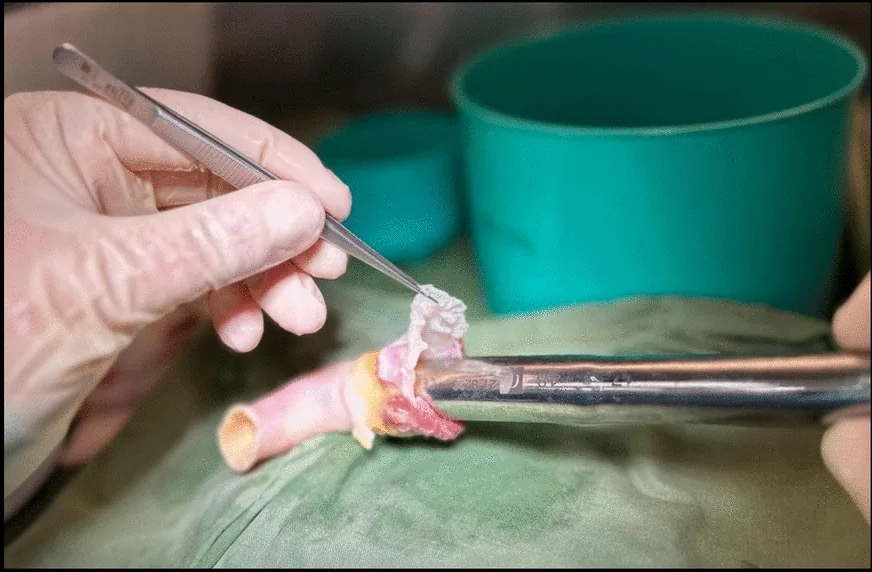
Heart valves measurements. Representative image of heart valve measurement during tissue processing

Tissues’ dimensions are measured (Fig. 5) and recorded, including annulus diameter and conduit length, to enable appropriate matching with recipient requirements.

Functional assessment is performed using a physiological saline test. The homograft is filled with solution under controlled pressure to evaluate valve competence and detect any leakage or insufficiency. Valves demonstrating structural defects, leakage, or abnormal morphology are discarded. All findings, including photographic documentation where applicable, are documented in standardized batch records, ensuring full traceability and compliance with regulatory requirements.

### Decontamination

Tissues are incubated in a commercial antibiotic cocktail (BASE.128, Alchimia) supplemented with 0.2 mg/ml Ciprofloxacin at 2–8 °C for 24–48 h.

### Cryopreservation and storage

Processed homografts are submerged in Medium-199 containing 10% dimethyl sulfoxide (DMSO) (WAK-Chemie Medical GmbH) and packaged in double-layered sterile EVA cryobags (Macropharma). Sealing is performed using a validated heat-sealing system. The packaging has been qualified to ensure maintenance of sterility and structural integrity throughout storage. Long-term routine use demonstrated consistent performance without failures in packaging integrity.

Controlled-rate freezing is performed using a programmable freezer (Planer Kryo 560), prior to storage in vapor-phase liquid nitrogen tanks (≤ − 150 °C). Storage temperature is continuously monitored with automated alarm systems in place.

### Quality assurance and tissue release

Grafts remain in quarantine until release is authorized by an external Quality Assurance (QA) based on sterility results, process documentation and personnel sampling as follows:

#### Sterility testing

In addition to the initial bioburden assessment described earlier, microbiological assessments of the final tissue product are performed, including mycobacterial, aerobic, anaerobic, and fungal cultures (using the BacT/ALERT and eSwab systems), as previously described (Levy et al. 2025). Tissue is considered unsuitable for transplantation and discarded if any of these cultures returns positive.

#### Quality assurance review

Each homograft is assigned a unique identifier code and accompanied by a folder for full traceability. A dedicated batch record is included, presenting donor screening data, processing timeline, temperature logs, sterility test results, personnel and environmental identifiers and microbiological monitoring.

Final product release is authorized only after completion of all required testing QA review. Tissues are approved for clinical use for up to five years, in accordance with validated storage protocols.

#### Distribution

Allograft allocation is coordinated through the Israel Transplantation Center, under the Ministry of Health (Fig. 1). Following approval, the allograft is transported in a Dry Shipper, a liquid nitrogen vapor container that maintains temperatures below − 150 °C (MVE Vapor Shipper). To validate and document safe shipping of the tissue, continuous temperature monitoring is performed using a calibrated PDF data logger from the time of removal from storage until delivery to the operating room. If the graft was not implanted, temperature monitoring and documentation continue until the graft is returned to the tissue bank’s storage.

## Results

### Cardiovascular tissue donors

Between January 2018 and April 2025, 142 donor hearts were retrieved and processed at the Sheba Cardiovascular Tissue Bank. The processed cardiovascular tissues included aortic homografts, pulmonary homografts, and pulmonary patches.

As shown in Table 1, the majority of the hearts came from deceased donors (92.3%), with a smaller proportion originating from living donors (7.7%).

**Table 1:** Cardiovascular tissue donors’ categories

Category	Number of donors	Percent
Cardiac	39	27.5
Cerebrovascular	42	29.6
Trauma	30	21.1
Suicide	20	14.1
Heart transplant recipients	11	7.7
Total	142	100.0

**Fig. 6:**
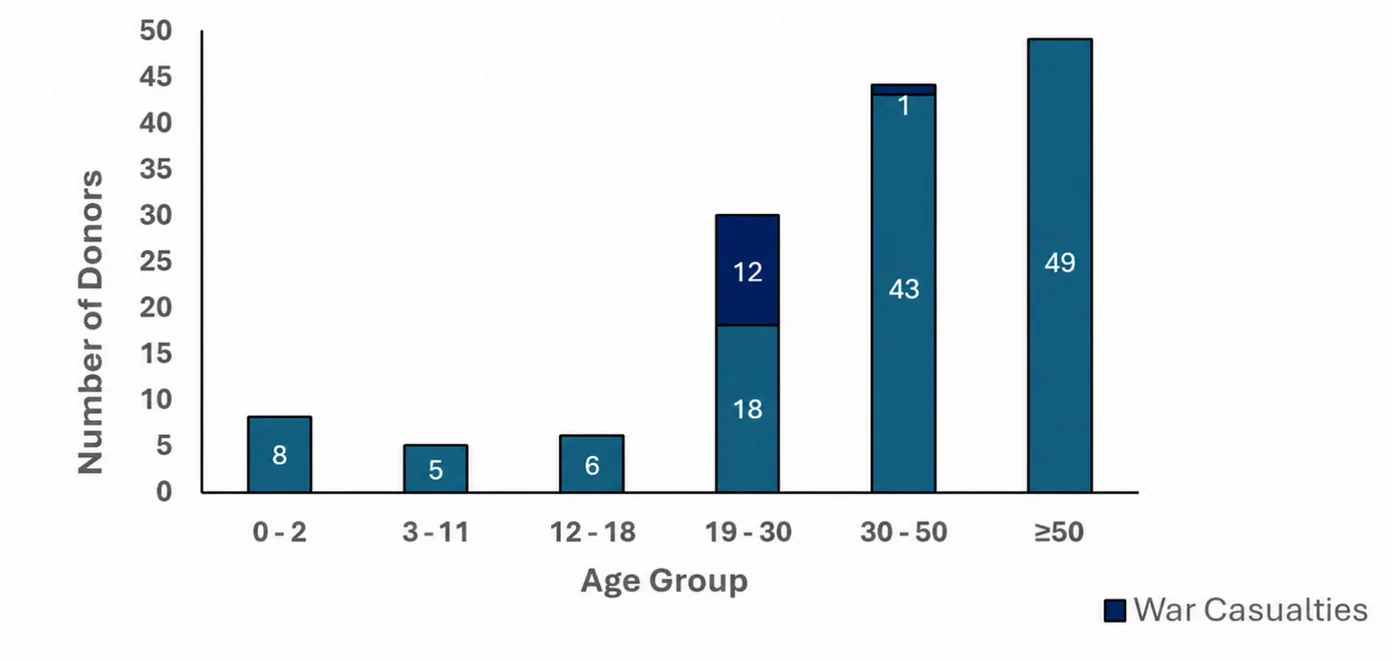
Cardiovascular tissue donors by age

The age distribution of donors, as depicted in Fig. 6, shows a clear trend toward increased heart donations from older individuals, particularly those aged 50 and older, accounting for 49 donors (35%) above the age of 50. The lowest number of donors was observed in the pediatric groups, with 8 donors (6%) aged 0–2 years, 5 donors (4%) aged 3–11 years, and 6 donors (4%) aged 12–18 years.

A moderate increase was noted in young adults (19–30 years), totaling 30 donors, 40% of whom were war casualties. This highlights the significant role of external circumstances, such as armed conflict, in shaping donor demographics. The causes of death among pediatric donors are heterogeneous and predominantly acute, with trauma emerging as a leading contribution (Table 2).

**Table 2:** Cause of death in pediatric population

Cause of Death	Age 0–2 years	Age 2–11 years	Age 12–18 years
Drowning	1	2	1
Road accident	2	–	–
Spontaneous hemorrhage/stroke	2	–	–
Sudden infant death syndrome	1	–	–
Trauma	1	1	2
Cardiac disease	–	1	–
Aspiration	1	–	1
Live donor – Heart transplant recipient	–	1	1
Suicide	–	–	1
Total	8	5	6

Overall, these findings indicate that the donor pool is predominantly composed of adults aged over 30, with a relatively limited contribution from younger age groups.

### Cardiovascular tissue retrieval and distribution trends

**Fig. 7:**
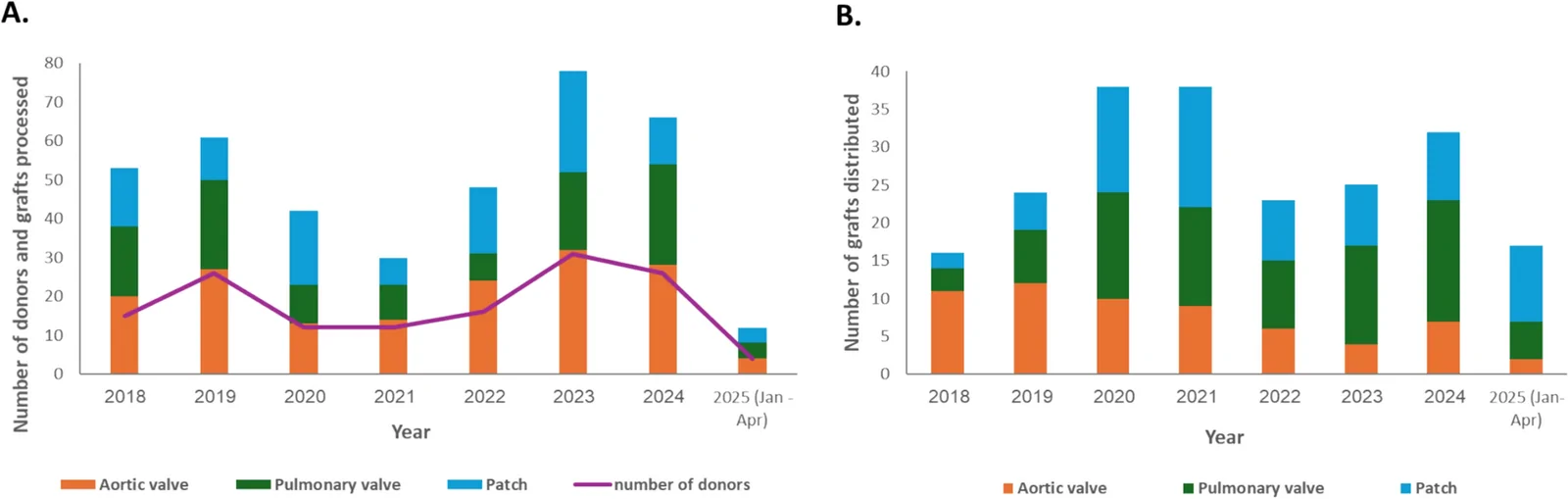
Sheba Cardiovascular Tissue Bank activity, 2018–2025. **A** Yearly number of cardiovascular tissue donors and cardiovascular tissues generated, including aortic homografts, pulmonary homografts, and pulmonary patches. **B** Yearly cardiovascular tissue distribution to hospitals nationwide by tissue type

The number of allografts processed at the bank fluctuated over the years in direct correlation with donor availability (Fig. 7A). After an initial growth in donations, a notable decline occurred in 2020–2021, attributable to the COVID-19 pandemic, which was associated with widespread disruption of health-care systems, as well as strict safety protocols, more stringent donor screening criteria, and logistical challenges. In contrast, 2023 marked a significant recovery and peak in retrieval and processing, likely driven by the ongoing regional conflict, which led to an increase in the number of deceased donors (16 in 2022 *vs.* 31 in 2023, of which 13 were battlefield casualties).

### Protocol refinement

We previously reported the detailed decontamination protocol (Levy et al. 2025). As reported, we found that transitioning from an in-house prepared antibiotics mix to a commercial antibiotic solution was followed by the emergence of *Mycobacterium simiae* detected in the final product, and the discarding of tissues. Following this observation, we currently supplement the commercial BASE.128 solution with Ciprofloxacin. Up to date, 13 cardiovascular tissues were treated with this solution, and none were found to be infected. In comparison, we previously reported that 12.3% cardiovascular tissues (9 out of 73 treated with BASE.128 alone) were discarded due to infection found in the final product (Levy et al. 2025).

### Cardiovascular tissue distribution

The cardiovascular tissues are distributed to cardiac surgery centers in Israel. Figure 7B presents the yearly distribution of cardiovascular tissue. An upward trend from 2018 through 2021 was followed by a decline in 2022, in which year the Ministry of Health, in accordance with the Declaration of Istanbul on Organ Trafficking and Transplant Tourism, forbade the distribution of tissues to non-Israeli citizens (Muller et al. 2019). Notably, prior to the introduction of this policy, more than half of the homografts were allocated to foreign citizens. All cardiovascular tissues described in this study were processed and distributed through the Israeli national tissue banking system. A gradual and constant increase from that year reflects the growing clinical reliance on locally processed cardiovascular tissues and the bank’s enhanced operational capacity.

### Cardiovascular tissue recipients

**Fig. 8:**
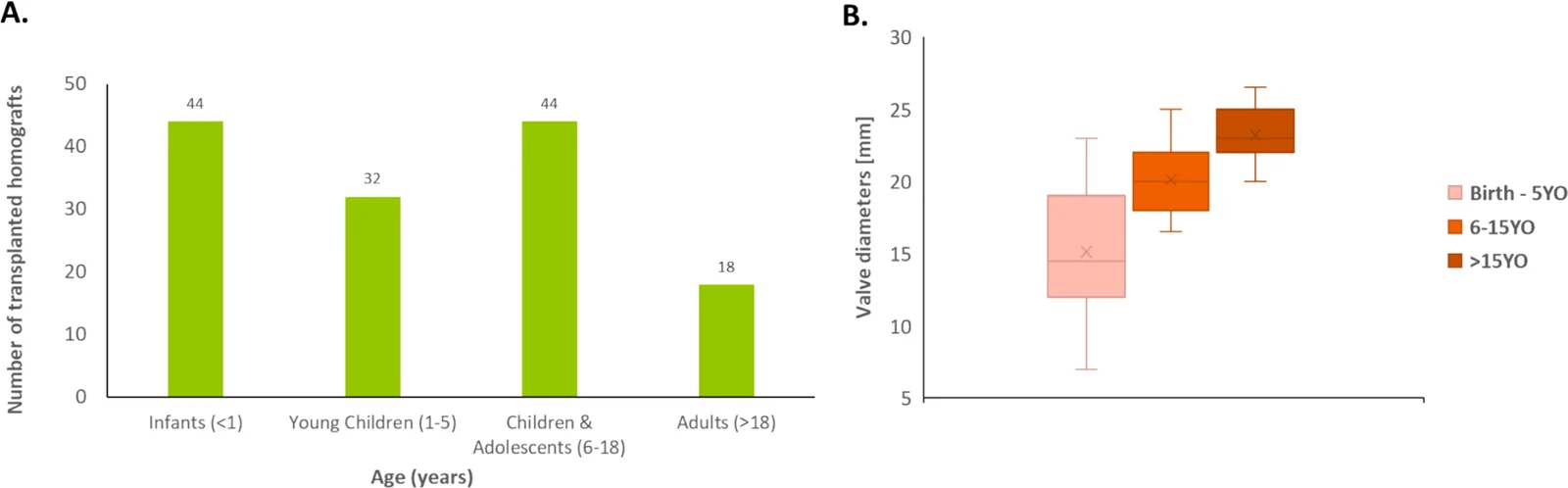
Cardiovascular tissue allocation by recipients’ age. **A** Number of grafts allocated by age groups. **B** Tissue diameters by recipient age group. YO – years old

Most of the grafts are allocated for pediatric patients undergoing complex congenital heart procedures, with 86% of the cardiovascular tissues distributed to patients in the age ranges of infancy to adolescence (birth-18 years old), including 31% allocated to infant patients (Fig. 8A).

The grafts range in diameter from 6 to 28 mm, accommodating the needs of a broad spectrum of pediatric and adult patients. Figure 8B illustrates the relation between recipient ages and the diameter of implant homografts. As expected, a positive correlation is observed between these two factors, underscoring the importance of offering a diverse selection of cardiovascular tissue sizes to ensure the best possible outcomes for all age groups.

**Fig. 9:**
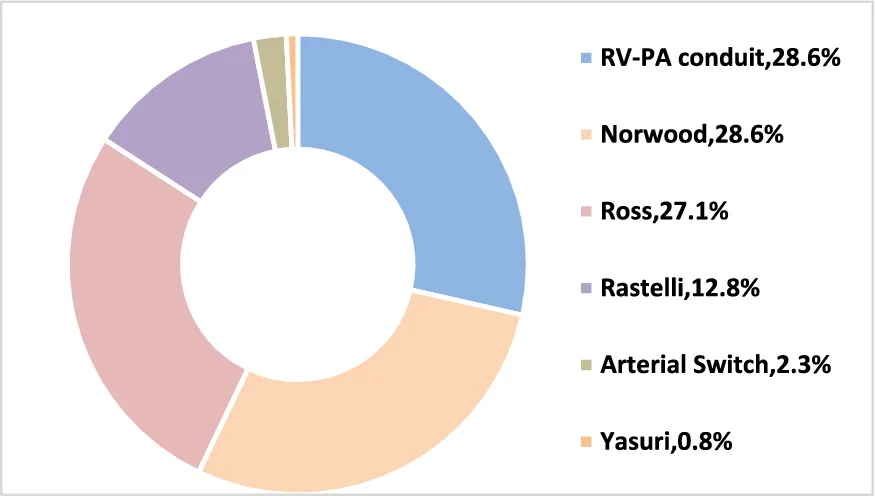
Cardiovascular tissue usage by surgery

Between 2018 and 2025, homografts were primarily employed in Norwood and right ventricle-to-pulmonary artery conduit reconstructions, each accounting for 28.6% of all applications. This underscores the essential role of homografts in the surgical management of complex congenital heart disease, particularly among neonatal and pediatric patients. The Ross procedure accounted for 27.1% of homograft use, highlighting its significance in acquired aortic valve pathology within this population. Remaining applications included Rastelli (12.8%), Arterial Switch (2.3%), and Yasui (0.8%) procedures (Fig. 9), collectively illustrating the procedural breadth for which homograft implantation is essential. Overall, these findings emphasize the enduring importance of homografts in addressing a diverse spectrum of complex cardiac surgeries throughout the study period.

## Discussion

The Sheba Cardiovascular Tissue Bank was established to meet the specific clinical needs of cardiac surgeons, particularly those treating congenital heart defects, who require timely access to high-quality human cardiovascular tissue grafts. Over seven years of operation, the bank has progressed from basic sterile processing to full GMP-compliant cleanroom procedures, demonstrating a sustainable and scalable model for public hospital-based cardiovascular tissue banking. The variation in patient ages and surgical procedures emphasizes the need to maintain a versatile inventory of cryopreserved cardiovascular tissues to accommodate the broad spectrum of surgical indications encountered in a diverse patient population.

Sheba Cardiovascular Tissue Bank framework and procedures align with those of European banks with slight changes. In general, tissues may be procured in hospitals or specialized tissue recovery centers (Castells-Sala et al. 2024; Golemovic et al. 2022; Jashari et al. 2023). In contrast, the Sheba Cardiovascular Tissue Bank team performs the procurement in any hospital within the country without transferring the body, which may account in part for the low discard rates. At the Sheba Cardiovascular Tissue Bank, the same trained tissue bank staff perform the entire workflow, from procurement to processing and distribution, which may enhance process continuity and facilitate unified quality control across all stages. Compared to major European centers such as the Barcelona Tissue Bank, which processed over 3000 homografts over two decades (Castells-Sala et al. 2024), the Sheba Cardiovascular Tissue Bank represents a small, yet highly efficient, program operating within a focused national framework.

A low discard rate characterizes Sheba’s cardiovascular tissue production, with only 13.3% of tissue discarded, primarily due to donor-related factors or positive cultures in the final product (Levy et al. 2025). The low discard rate may reflect the comprehensive donor evaluation process, standardized tissue assessment procedures, GMP-compliant processing conditions, external quality assurance review, and continuous monitoring of procurement, manufacturing, and distribution processes described in this study.

An example of constant process review is the decontamination protocol alteration implemented after identifying increased discard rates with the commercial BASE.128 solution alone; supplementation with ciprofloxacin subsequently eliminated infections in treated tissues, and ongoing monitoring will determine if further modifications are required. Similarly, we recently published a process review of the musculoskeletal (MSK) tissue division’s procurement protocol, which was revised in response to elevated contamination rates in field-casualty tissues (Cohen et al. 2026). These improvements are facilitated by the integrated tissue bank operation, where the same staff, trained across the entire workflow from procurement to distribution, enables seamless quality evaluation and protocol refinement.

### Challenges and future directions

One of the critical challenges facing the Sheba Cardiovascular Tissue Bank is the limited number of tissue donors, specifically infants, relative to clinical demand. Organ and tissue donations in Israel follow an ‘opt-in’ model, where the final authority rests with the next of kin, even in instances where a deceased carries an official organ donor card. This requirement may negatively impact the volume of available tissues for banking (Molina-Pérez et al. 2022). Expanding the donor base is essential to ensure long-term sustainability, particularly for pediatric patients who require size-matched grafts, and should entail a multifaceted strategy that includes public education initiatives, streamlined hospital donor identification systems, and robust digital tracking infrastructure. Nevertheless, a wide range of homografts’ size is supplied to answer the needs of infant recipients, and the inability to meet a surgeon’s demand is a rare occurrence.

Currently, the Sheba Cardiovascular Tissue Bank is actively working on the development of an in-house decellularization protocol for human heart valves. This process aims to remove all viable donor cells while preserving the extracellular matrix, potentially reducing immunogenicity and enabling better graft integration through host cell repopulation. Although the protocol is still under development and has not yet been applied clinically, this line of research represents a significant step toward reducing graft immunogenicity and improving tissue integration, particularly for pediatric patients and those with heightened immunological risk. The successful validation of such a protocol would position the bank at the forefront of evolving regenerative tissue engineering practices.

Other challenges remain, including implementing digital platforms for real-time updates on graft inventory and utilization tracking. Patient follow-up is another challenge that needs to be addressed, especially for research and evaluation purposes. Nonetheless, the Sheba Cardiovascular Tissue Bank model demonstrates that, with institutional commitment and strong clinical collaboration, a high-quality cardiovascular tissue bank that can operate successfully at a national level, even within a limited-volume setting.

## Data Availability

The datasets generated during and/or analysed during the current study are available from the corresponding author upon reasonable request.
